# Supplementary material for: The Relationship between Physical Activity and Long COVID: A Cross-Sectional Study
Source: Int J Environ Res Public Health. 2022 Apr 22;19(9):5093. doi: 10.3390/ijerph19095093 (PMC9105041; doi:10.3390/ijerph19095093)
Supplement: Supplementary file 1 [file ijerph-19-05093-s001.zip › Online survey supp file_V2.pdf]

Supplementary material – copy of online survey without logic

PA and long covid

Page 1: Participant information and data privacy notice

Understanding the Relationship Between Physical Activity and Long Covid

Please note this questionnaire is only for individuals who **currently** have symptoms of long covid.

The purpose of this research study is to get your personal experience on how different types of physical activity affects your long covid symptoms. The following questionnaire will take you approximately 15 minutes to complete.

If you are unable to complete the survey in one go you can save your answers by selecting the 'finish later' button at the end of each page and following the instructions. However, to be included in the research project you **must** complete the questionnaire within a 24 hour period.

As a follow on to this questionnaire, we may invite you for an interview at a later date. However, this is **optional** and you can complete the online survey without being interviewed.

Please read the university privacy notice and participant information sheet below prior to completing the questionnaire. We advise downloading a copy of these for your records. If you have any questions prior to taking part please contact us at bs20j2w@leeds.ac.uk

- University of Leeds privacy notice
- Participant information sheet

1. To proceed to the questionnaire please tick the boxes below *Required*

- ☐ I confirm that I have read the information above, including the participant information sheet, and understand what I am being asked to do in this research.
- ☐ I understand that my participation is voluntary and that I am free to withdraw at any time without giving any reason and without there being any negative consequences. However, I understand that once I have submitted my answers to the online survey they cannot be withdrawn from the study. If I am invited for interview, I understand I will have two weeks after the interview to withdraw my data from the study. In addition, should I not wish to answer any particular question or questions, I am free to decline.
- ☐ I give permission for members of the research team to have access to my anonymised responses. I understand that my name will not be linked with the research materials, and I will not be identified or identifiable in the report or reports that result from the research.
- ☐ I agree for the data collected from me to be stored and used in relevant future research in an anonymised form.
- ☐ Should I have an interview as a follow on to this survey, I give permission for direct quotes from the interview to be used and understand that they will remain anonymous.
- ☐ I agree to take part in the above research project.

Page 2: About you

2. How old are you? (please give your answer in years and months)

3. What is your sex?

More info

- Female
- Male
- I'd prefer not to say

4. Please select the answer that best describes your ethnicity

More info

- White (British, Irish, Irish Traveller or other White backgrounds)
- Black (African, Caribbean or other Black backgrounds)
- Asian (Indian, Pakistani, Bangladeshi, Chinese or other Asian backgrounds)
- Mixed (White and Asian, White and Black African, White and Black Caribbean, Other)
- Other
- I'd prefer not to say

4.a. If you selected Other, please specify:

5. What is your height? (in cm **or** feet and inches)

	centimeters	feet and inches	I'm not sure

height			

6. What is your weight? (in kg **or** stones and pounds)

	kilogrammes	stones and pounds	I'm not sure
weight			

7. What is the first part of your postcode? (e.g. LS8)

8. Regarding your health, please tick all of the following apply to you

- Pregnancy
- Diabetes (type 1 or 2)
- Chronic respiratory conditions (e.g. asthma, COPD)
- Chronic heart disease (e.g. ischaemic heart disease, heart failure)
- Chronic kidney disease
- Chronic liver disease
- Chronic neurological conditions (e.g. Parkinson's disease, MS)
- Cancer
- Autoimmune diseases (e.g. Crohn's disease, ulcerative colitis, rheumatoid arthritis, systemic lupus)
- Other
- I do not have any medical conditions
- I'd prefer not to say

8.a. If you selected Other, please specify:

Page 3: COVID-19

9. When did you first have COVID-19 symptoms?

Dates need to be in the format 'DD/MM/YYYY', for example 27/03/1980.

(dd/mm/yyyy)

10. Was your COVID-19 infection confirmed?

- ☐ Yes, by a PCR test (throat and nose swab)
- ☐ Yes, by an antibody blood test
- ☐ Yes, but it was based on my symptoms alone
- ☐ Other
- ☐ I'd prefer not to say

10.a. If you selected Other, please specify:

11. Please select all the symptoms you've experienced in **the last 7 days** (tick all that apply)

- ☐ Abdominal pain
- ☐ Brain fog, loss of concentration or memory issues
- ☐ Breathlessness
- ☐ Chest pain
- ☐ Chest tightness
- ☐ Confusion
- ☐ Cough
- ☐ Diarrhoea
- ☐ Dizziness
- ☐ Earache
- ☐ Fatigue
- ☐ Fever
- ☐ Headache

- ☐ Joint pain
- ☐ Loss of taste and/or smell
- ☐ Muscle pain
- ☐ Nausea
- ☐ Pain
- ☐ Palpitations
- ☐ Post-exertional malaise
- ☐ Reduced appetite
- ☐ Sensation of pins and needles or numbness
- ☐ Skin rashes
- ☐ Sleep disturbance
- ☐ Sore throat
- ☐ Symptoms of anxiety
- ☐ Symptoms of depression
- ☐ Tinnitus (ringing in your ears)
- ☐ Other
- ☐ I'd prefer not to say

11.a. If you selected Other, please specify:

Page 4: Activities of Daily Living

Questions 12-15 are about activities of daily living. Activities of daily living are simple self-care tasks you do around the home.

Examples include:

- **Personal hygiene – bathing/showering, grooming, nail care, and oral care.**
- **Dressing - being able to make appropriate clothing decisions and physically dress and undress oneself.**
- **Eating - the ability to feed oneself, though not necessarily the capability to prepare food.**
- **Maintaining continence - being able to mentally and physically use a restroom. This includes the ability to get on and off the toilet and cleaning oneself.**
- **Transferring/Mobility- being able to stand from a sitting position, as well as get in and out of bed. The ability to walk independently from one location to another.**

12. **Before** you had COVID-19, how many days per week would you need help with and/or not manage to complete any activity of daily living?

- 0 days
- 1 day
- 2 days
- 3 days
- 4 days
- 5 days
- 6 days
- 7 days

13. In **the last 7 days**, how many days have you needed help with and/or not managed to complete any activity of daily living?

- 0 days
- 1 day
- 2 days
- 3 days

- 4 days
- 5 days
- 6 days
- 7 days

14. If there has been a change in your ability to complete any activity of daily living, how much of this is because of yourlong covid symptoms?
0% = my symptoms have nothing to do with my change in activities of daily living (it's changed for other reasons). 100% = my symptoms are the only reason my activities of daily living levels have changed

Please don't select more than 1 answer(s) per row.

	0-20%	20-40%	40-60%	60-80%	80-100%
change in activities of daily living due to long covid	☐	☐	☐	☐	☐

15. In **the last 7 days**, has completing activities of daily living changed any of your long covid symptoms?

- Yes, it's made my symptoms worse
- Yes, it's made my symptoms better
- Yes, sometimes it makes my symptoms better but sometimes it makes them worse
- No

15.a. For symptoms that are worsened by activities of daily living, is this during or after the activity?

- During
- After
- Both

15.b. In **the last 7 days**, which symptoms have been worsened by completing activities of daily living? (please list all the symptoms this applies to)

15.c. For symptoms that are improved activities of daily living, is this during or after the activity?

- ○ During
- ○ After
- ○ Both

15.d. In **the last 7 days**, which symptoms have been improved by completing activities of daily living? (please list all the symptoms this applies to)

Page 5: Brisk Walking

Questions 16 & 17 are about walking at a brisk pace, this is a pace at which you are breathing harder than normal.

Examples include:

- **Walking at work or school**
- **Walking to get from place to place**
- **Walking at home or any activities that you did solely for recreation, sport, exercise or leisure**

16. **Before** you had COVID-19, how many days per week would you walk at a brisk pace?

- ○ 0 days
- ○ 1 day
- ○ 2 days
- ○ 3 days
- ○ 4 days
- ○ 5 days
- ○ 6 days
- ○ 7 days

16.a. On average, how long did you spend walking at a brisk pace on each of those days? (please give your answer in minutes)

Please enter a whole number (integer).

17. In **the last 7 days,** how many days have you walked at a brisk pace?

- ○ 0 days
- ○ 1 day
- ○ 2 days

- 3 days
- 4 days
- 5 days
- 6 days
- 7 days

17.a. On average, how long did you spend walking at a brisk pace on each of those days? (please give your answer in minutes)

Please enter a whole number (integer).

17.b. If there has been a change in the amount of brisk walking you've been doing, how much of this is because of your long covid symptoms?
0% = my symptoms have nothing to do with my change in brisk walking levels (it's changed for other reasons). 100% = my symptoms are the only reason my brisk walking levels have changed

Please don't select more than 1 answer(s) per row.

	0-20%	20-40%	40-60%	60-80%	80-100%
change brisk walking due to long covid	☐	☐	☐	☐	☐

17.c. In **the last 7 days**, has brisk walking changed any of your long covid symptoms?

- Yes, it's made my symptoms worse
- Yes, it's made my symptoms better
- Yes, sometimes it makes my symptoms better but sometimes it makes them worse
- No

17.c.i. For symptoms that are worsened by brisk walking, is this during or after the activity?

- During
- After
- Both

17.c.ii. In **the last 7 days**, which symptoms have been worsened by brisk walking? (please list all the symptoms this applies to)

17.c.iii. For symptoms that are improved by brisk walking, is this during or after the activity?

- ○ During
- ○ After
- ○ Both

17.c.iv. In **the last 7 days**, which symptoms have been improved by brisk walking? (please list all the symptoms this applies to)

Page 6: Moderate Physical Activity

Questions 18 & 19 are about moderate physical activity, these activities make you breathe harder than normal, but only a little. Please don't include any walking here.

Examples include:

- **Carrying light loads**
- **Cycling at a regular pace**
- **Heavy gardening (digging, weeding, raking, planting, pruning, clearing section)**
- **Heavy cleaning (sweeping, cleaning windows, moving furniture)**
- **DIY**

18. **Before** you had COVID-19, how many days per week did you do moderate physical activity?

- ○ 0 days
- ○ 1 day
- ○ 2 days
- ○ 3 days
- ○ 4 days
- ○ 5 days
- ○ 6 days
- ○ 7 days

18.a. On average, how long did you spend doing moderate physical activity on each of those days? (in minutes)

Please enter a whole number (integer).

[ ]

19. In **the last 7 days,** how many days have you done moderate physical activity?

- 0 days
- 1 day
- 2 days
- 3 days
- 4 days
- 5 days
- 6 days
- 7 days

19.a. On average, how long did you spend doing moderate physical activity on each of those days? (in minutes)

Please enter a whole number (integer).

19.b. If there has been a change in the amount of moderate physical activity you've been doing, how much of this is because of your long covid symptoms?
0% = my symptoms have nothing to do with my change in moderate activity levels (they've changed for other reasons). 100% = my symptoms are the only reason my moderate activity levels have changed

Please don't select more than 1 answer(s) per row.

	0-20%	20-40%	40-60%	60-80%	80-100%
change moderate activities due to long covid	☐	☐	☐	☐	☐

19.c. In **the last 7 days**, has moderate physical activity changed any of your long covid symptoms?

- Yes, it's made my symptoms worse
- Yes, it's made my symptoms better
- Yes, sometimes it makes my symptoms better but sometimes it makes them worse
- No

19.c.i. For symptoms that are worsened by moderate physical activity, is this during or after the activity?

- During
- After
- Both

19.c.ii. In **the last 7 days**, which symptoms have been worsened by moderate physical activity? (please list all the symptoms this applies to)

19.c.iii. For symptoms that are improved by moderate physical activity, is this during or after the activity?

- During
- After
- Both

19.c.iv. In **the last 7 days**, which symptoms have been improved by moderate physical activity? (please list all the symptoms this applies to)

Page 7: Vigorous Physical Activity

Questions 20 & 21 are about vigorous physical activity, these activities make you breathe a lot harder than normal ('huff and puff'). Please don't include any walking here.

Examples include:

- **Heavy lifting**
- **Fast cycling**
- **Exercise classes**
- **Fast swimming**
- **Sports like football or basketball**

20. **Before** you had COVID-19, how many days per week did you do vigorous physical activity?

- 0 days
- 1 day
- 2 days
- 3 days
- 4 days
- 5 days
- 6 days
- 7 days

20.a. On average, how long did you spend doing vigorous physical activity on each of those days? (in minutes)

Please enter a whole number (integer).

21. In **the last 7 days,** how many days have you done vigorous physical activity?

- 0 days
- 1 day
- 2 days
- 3 days
- 4 days
- 5 days
- 6 days
- 7 days

21.a. On average, how long did you spend doing vigorous physical activity on each of those days? (in minutes)

Please enter a whole number (integer).

21.b. If there has been a change in the amount of vigorous physical activity you've been doing, how much of this is because of your long covid symptoms?
0% = my symptoms have nothing to do with my change in vigorous activity levels (they've changed for other reasons). 100% = my symptoms are the only reason my vigorous activity levels have changed

Please don't select more than 1 answer(s) per row.

	0-20%	20-40%	40-60%	60-80%	80-100%
change vigorous activities due to long covid	☐	☐	☐	☐	☐

21.c. In **the last 7 days**, has vigorous physical activity changed any of your long covid symptoms?

- Yes, it's made my symptoms worse
- Yes, it's made my symptoms better
- Yes, sometimes it makes my symptoms better but sometimes it makes them worse
- No

21.c.i. For symptoms that are worsened by vigorous physical activity, is this during or after the activity?

- During
- After
- Both

21.c.ii. In **the last 7 days**, which symptoms have been worsened by vigorous physical activity? (please list all the symptoms this applies to)

21.c.iii. For symptoms that are improved by vigorous physical activity, is this during or after the activity?

- During
- After
- Both

21.c.iv. In **the last 7 days**, which symptoms have been improved by vigorous physical activity? (please list all the symptoms this applies to)

Page 8: Physical Activity and Long Covid

22. Have you been diagnosed with any medical complications as a consequence of being physically active with long covid?

- ◯ Yes
- ◯ No

22.a. Please list the complications you've experienced

23. Did a health care professional (e.g. doctor, physio, nurse) recommend physical activity as part of your long covid recovery?

- ◯ Yes
- ◯ No

23.a. What type of physical activity did they recommend?

23.b. Did you read or hear anywhere else that physical activity was beneficial for long covid recovery?

- ☐ Friends
- ☐ Social media or blog posts
- ☐ Academic journals or publications
- ☐ Other
- ☐ I wasn't advised by anyone but thought it might help
- ☐ No

23.b.i. If you selected Other, please specify:

Graded exercise therapy is where you continuously increase the amount or intensity of exercise week by week, irrespective of whether your symptoms are getting better or worse.

Pacing is where the level of physical activity you do on a particular day is based on how your symptoms are on that day. If you are having a good day you might do a little extra or push yourself a little harder, but you'd still work within your limits.

24. Have you tried one of these strategies as part of your long covid recovery?

- ☐ Graded exercise therapy
- ☐ Pacing
- ☐ Other (I've tried a different strategy)
- ☐ None of the above

24.a. If you selected Other, please specify:

25. Did a health care professional (e.g. doctor, physio, nurse) tell you to avoid physical activity as part of your long covid recovery?

- ○ Yes
- ○ No

25.a. Did you read or hear anywhere else that physical activity should be avoided in long covid recovery?

- ☐ Friends
- ☐ Social media or blog posts
- ☐ Academic journals or publications
- ☐ Other
- ☐ I wasn't advised by anyone but thought this myself
- ☐ No

25.a.i. If you selected Other, please specify:

Page 9: Before you finish

26. Is there anything else about physical activity and long covid that you'd like to tell us about?

As a follow on to this questionnaire we will be conducting interviews (over Microsoft Teams or telephone) to learn more about physical activity and Long Covid. It will consist of a one-on-one interview lasting approximately 30 minutes.

27. Please leave a contact email address or telephone number below if you consent to taking part in an interview as part of this research project (don't worry, you can always change your mind at a later date). If you do not wish to be interviewed, you can simply ignore this question.

Page 10: Thank you

Thank you for your time, for filling out this questionnaire and helping us with our research.

If you have found completing any of these questions upsetting and you would like to speak to someone about your health or wellbeing please speak to your GP. Alternatively, you can talk to the Samaritans at Samaritans.org or call 116 123.

For information about COVID-19, please visit the following websites:

- www.gov.uk/coronavirus
- www.nhs.uk/conditions/coronavirus-covid-19/
- www.yourcovidrecovery.nhs.uk/
