# Supplementary material for: The Relationship between Physical Activity and Long COVID: A Cross-Sectional Study
Source: Int J Environ Res Public Health. 2022 Apr 22;19(9):5093. doi: 10.3390/ijerph19095093 (PMC9105041; doi:10.3390/ijerph19095093)
Supplement: Supplementary file 1 [file ijerph-19-05093-s001.zip › PA_and_LC_supplemental_material_V2_20.4.22.pdf]

Supplementary material

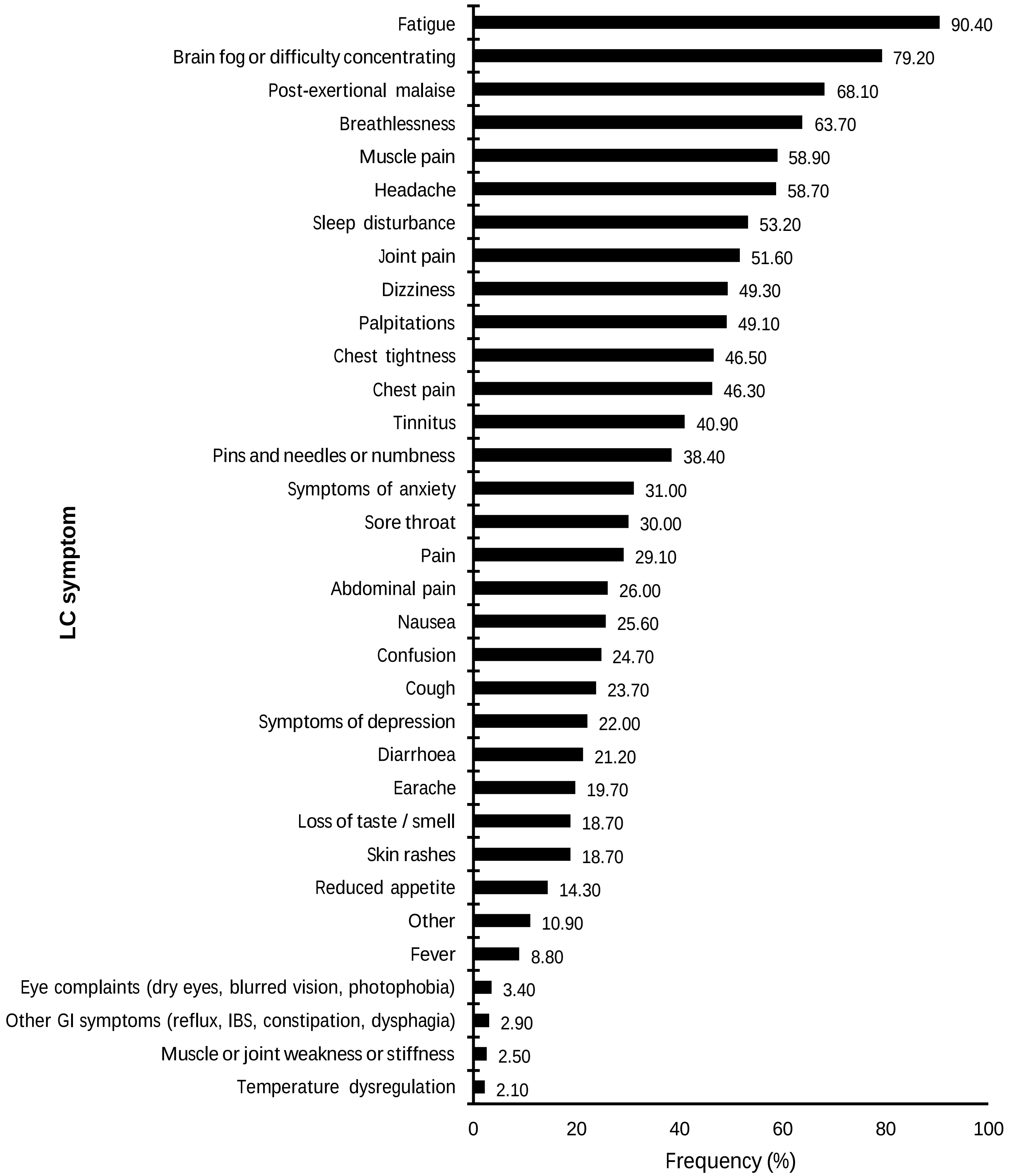

Figure S1. Frequency (%) of Long Covid (LC) symptoms in the last 7 days. Other includes all symptoms with a frequency of <2%.

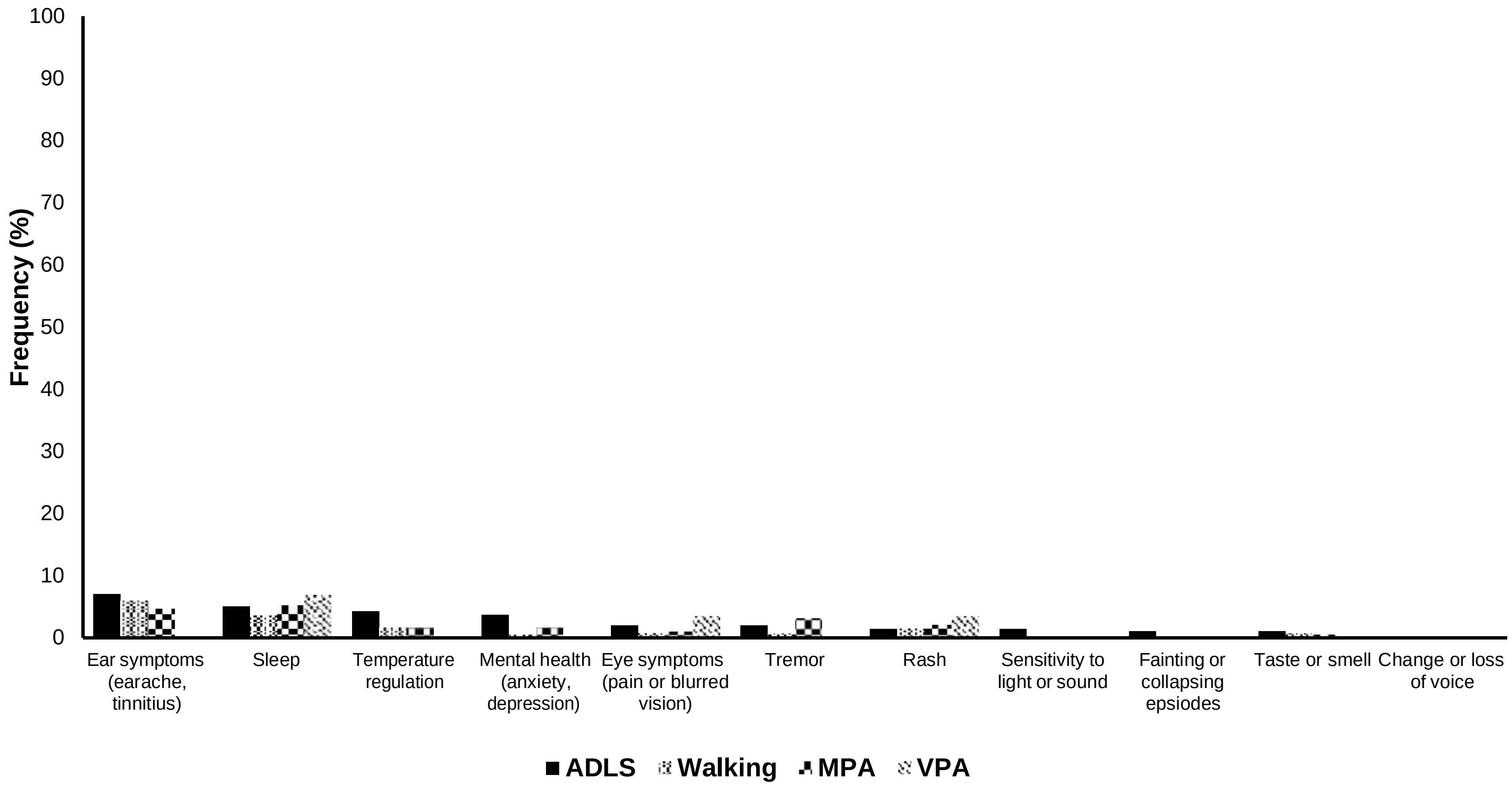

Figure S2. The frequency (%) of the remaining LC symptoms that are worsened by activities of daily living (ADL, n=353), brisk walking (n=133), moderate physical activity (MPA, n=189) and vigorous physical activity (VPA, n=29).

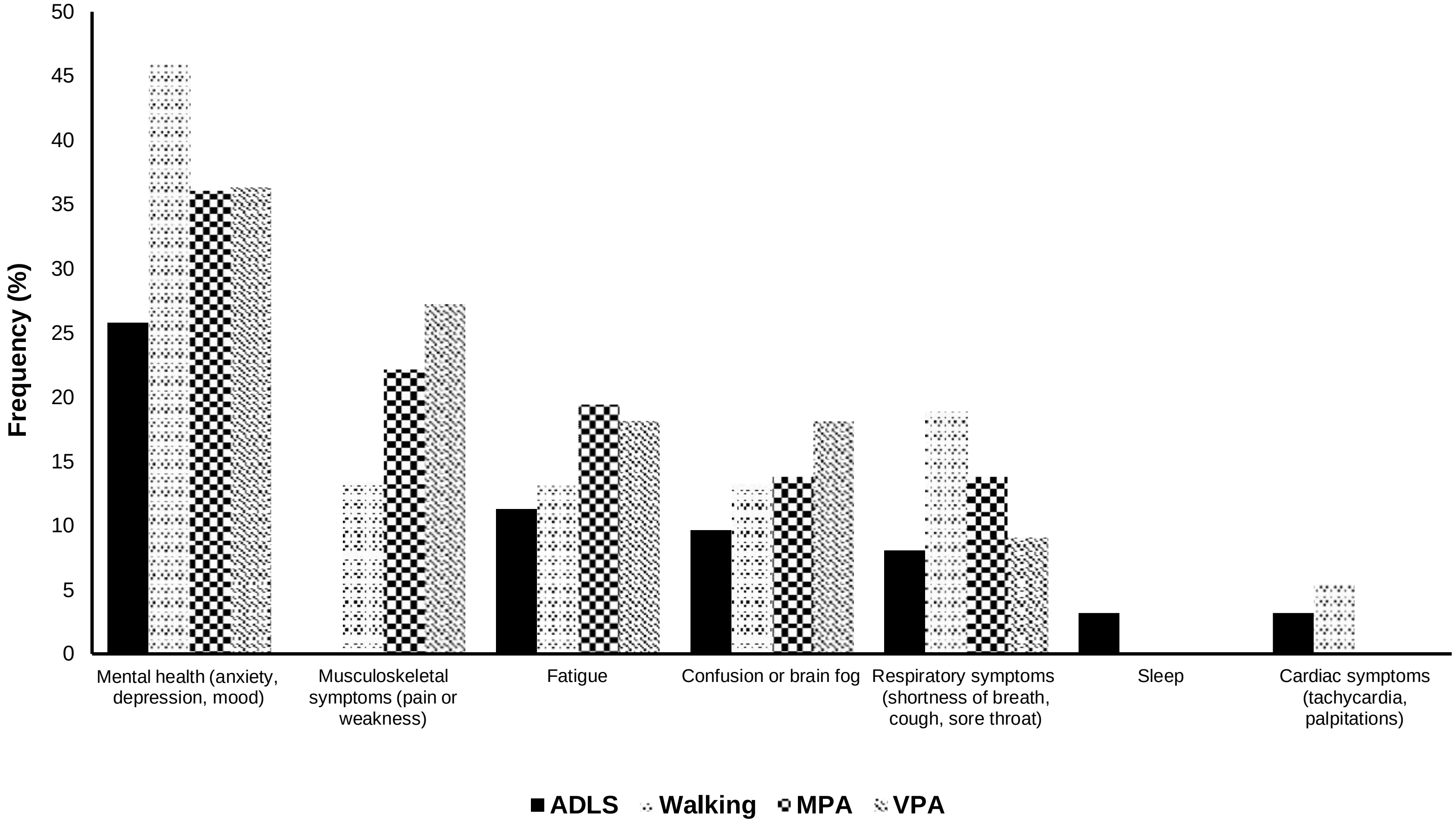

Figure S3a. The frequency (%) of LC symptoms that are improved by activities of daily living (ADL, n=62), brisk walking (n=37), moderate physical activity (MPA, n=36) and vigorous physical activity (VPA, n=11).

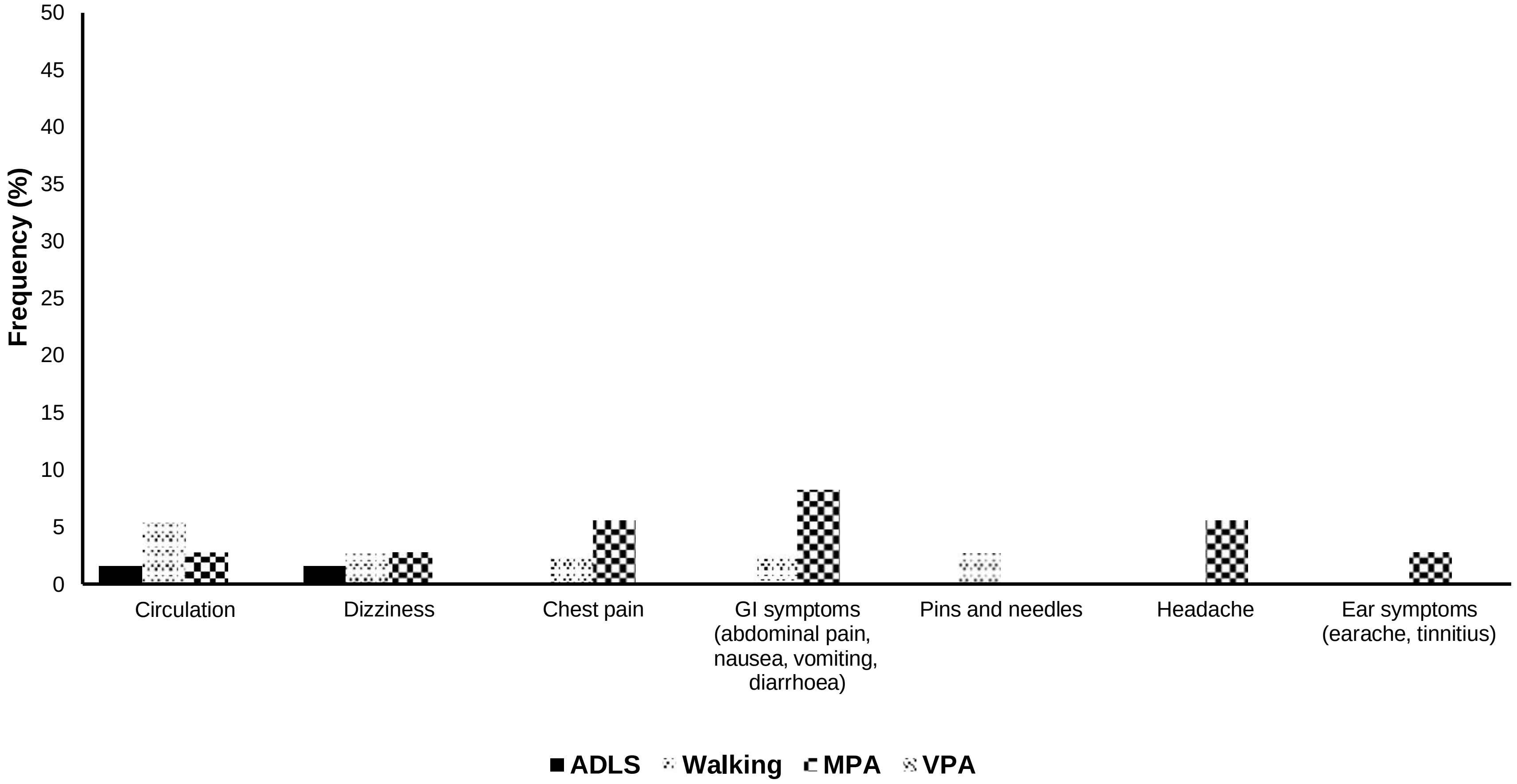

Figure S3b. The frequency (%) of LC symptoms that are improved by activities of daily living (ADL, n=62), brisk walking (n=37), moderate physical activity (MPA, n=36) and vigorous physical activity (VPA, n=11).

Table S1. Participant characteristics for each effect group of physical activity (PA) on Long Covid symptoms (LC) symptoms.

Characteristic	ADLs (n=468)		
	Worsened (n=291)	Mixed† (n=62)	No effect (n=115)
**Age (years), mean (SD)**	44.87 (9.76)	46.27 (11.57)	47.52 (9.72)
**Number of LC symptoms in the last 7 days, median (IQR)**	12.00 (9.00, 15.00)	10.00 (8.00, 15.00)	8.00 (5.00,11.00)
**Gender (female), n (%)**	273 (93.81)	52 (83.87)	93 (80.87)
**Time since COVID-19 symptom onset (months), n (%)**			
0-6	80 (27.97)	21 (34.43)	29 (25.44)
6-12	57 (19.93)	13 (21.31)	18 (15.80)
12-18	286 (52.10)	27 (44.26)	67 (58.77)
**Met UK PA guidelines pre-COVID-19, n (%)**	244 (83.85)	50 (80.64)	98 (85.22)
**Met UK PA guidelines post-COVID-19, n (%)**	17 (5.84)	9 (14.52)	13 (11.30)

Characteristic	Brisk walking (n=169)			
	Worsened (n=98)	Improved (n=2)	Mixed† (n=35)	No effect (n=34)
**Age (years), mean (SD)**	44.31 (8.62)	48.25 (12.37)	44.99 (11.35)	44.56 (13.11)

**Number of LC symptoms in the last 7 days, median (IQR)**	11.00 (8.00, 14.00)	10.00 (8.00, 12.00)	10.00 (7.00, 14.00)	7.00 (5.00 10.25)
**Gender (female), n (%)**	89 (90.82)	1 (50.00)	27 (77.14)	23 (67.64)
**Time since COVID-19 symptom onset (months), n (%)**				
0-6	24 (25.00)	0 (0.00)	9 (26.47)	9 (29.03)
6-12	19 (19.79)	1 (50.00)	8 (23.53)	5 (16.13)
12-18	53 (55.21)	1 (50.00)	17 (50.00)	17 (54.84)
**Met UK PA guidelines pre-COVID-19, n (%)**	83 (84.69)	1 (50.00)	28 (80.00)	27 (79.41)
**Met UK PA guidelines post-COVID-19, n (%)**	14 (14.29)	1 (50.00)	7 (22.86)	8 (23.53)

**Characteristic**	**MPA (n=223)**			
	Worsened (n=156)	Improved (n=3)	Mixed[†] (n=33)	No effect (n=31)
**Age (years), mean (SD)**	45.71 (9.39)	52.30 (9.91)	49.41 (9.03)	48.81 (13.73)
**Number of LC symptoms in the last 7 days, median (IQR)**	11.50 (8.00, 15.00)	8.00 (4.50, 9.00)	9.00 (7.00, 12.00)	6.00 (5.00, 8.00)

**Gender (female), n (%)**	147 (94.23)	0 (0.00)	28 (84.85)	25 (80.65)
**Time since COVID-19 symptom onset (months), n (%)**				
0-6	54 (35.53)	0 (0.00)	8 (24.24)	11 (36.67)
6-12	25 (16.45)	0 (0.00)	5 (15.15)	6 (20.00)
12-18	73 (48.03)	3 (100.00)	20 (60.61)	13 (43.33)
**Met UK PA guidelines pre-COVID-19, n (%)**	134 (85.90)	3 (100.00)	32 (96.97)	24 (77.42)
**Met UK PA guidelines post-COVID-19, n (%)**	22 (14.10)	1 (33.33)	10 (30.31)	5 (16.13)

**Characteristic**	**VPA (n=468)**		
	Worsened (n=18)	Mixed[†] (n=11)	No effect (n=4)
**Age (years), mean (SD)**	46.04 (8.30)	50.26 (11.38)	49.67 (15.68)
**Number of LC symptoms in the last 7 days, median (IQR)**	12.00 (7.75, 16.75)	9.00 (5.00, 15.00)	6.50 (3.75, 7.00)
**Gender (female), n (%)**	17 (94.44)	10 (90.91)	2 (50.00)
**Time since COVID-19 symptom onset (months), n (%)**			
0-6	7 (38.89)	4 (36.36)	1 (25.00)
6-12	2 (11.11)	2 (18.18)	1 (25.00)

12-18	9 (50.00)	5 (45.45)	2 (50.00)
**Met UK PA guidelines pre-COVID-19, n (%)**	17 (94.44)	10 (90.91)	2 (50.00)
**Met UK PA guidelines-post-COVID-19, n (%)**	9 (50.00)	10 (90.91)	0 (0.00)

ADL's; activities of daily living, IQR; interquartile range, n; number, MPA; moderate physical activity, PA; physical activity, SD; standard deviation, UK; United Kingdom, VPA; vigorous physical activity. †*mixed denotes that PA can both improve and worsen participants LC symptoms.*
